# Reflections on Health Promotion and Disability in Low and Middle-Income Countries: Case Study of Parent-Support Programmes for Children with Congenital Zika Syndrome

**DOI:** 10.3390/ijerph15030514

**Published:** 2018-03-14

**Authors:** Hannah Kuper, Tracey Smythe, Antony Duttine

**Affiliations:** International Centre for Evidence in Disability, London School of Hygiene & Tropical Medicine, London WC1E7HT, UK; tracey.smythe@lshtm.ac.uk (T.S.); antony.duttine@lshtm.ac.uk (A.D.)

**Keywords:** disability, health promotion, Zika, parent-support, low and middle income

## Abstract

Universal health coverage (UHC) has been adopted by many countries as a national target for 2030. People with disabilities need to be included within efforts towards UHC, as they are a large group making up 15% of the world’s population and are more vulnerable to poor health. UHC focuses both on covering the whole population as well as providing all the services needed and must include an emphasis on health promotion, as well as disease treatment and cure. Health promotion often focusses on tackling individual behaviours, such as encouraging exercise or good nutrition. However, these activities are insufficient to improve health without additional efforts to address poverty and inequality, which are the underlying drivers of poor health. In this article, we identify common challenges, opportunities and examples for health promotion for people with disabilities, looking at both individual behaviour change as well as addressing the drivers of poor health. We present a case study of a carer support programme for parents of children with Congenital Zika Syndrome in Brazil as an example of a holistic programme for health promotion. This programme operates both through improving skills of caregivers to address the health needs of their child and tackling poverty and exclusion.

## 1. Introduction

People with disabilities include those who have long-term physical, mental, intellectual or sensory impairments which in interaction with various barriers may hinder their full and effective participation in society on an equal basis with others (Table 1). The WHO estimates that there are one billion people living with disabilities globally, of whom 80% live in low and middle-income countries (LMICs) [1]. People with disabilities are more likely to be poor [2] and also face a broad range of exclusions, including from school, employment and social engagement [1]. These exclusions are inter-linked and reinforcing, for instance, a lack of money can reduce inclusion in school and lack of education can make it more difficult to find a job, which leads to deepening poverty. Disability has therefore increasingly become a focus in international development, because the number of people with disabilities is large and they are being left behind while progress is made for other groups. An example is the specific reference to disability within the Sustainable Development Goals (SDGs) adopted internationally in 2015, including with respect to Goals on “Quality education” and “Decent work” [3].

## 2. Health, Universal Health Coverage and Disability

Disability is not mentioned within the SDG for “Good Health and Well-Being”, which includes the target to “Achieve Universal Health Coverage” (UHC). UHC focuses on covering the whole population with the health services they need, without suffering financial hardship. These health services cover the full spectrum from promotion, treatment and rehabilitation to palliative care. There is a clear rationale for focusing on people with disabilities in this Goal and in efforts towards UHC, as there is growing evidence that people with disabilities are more at risk of experiencing poor health (e.g., non-communicable diseases, hypertension and mental health conditions) [4,5,6,7,8,9,10,11,12,13] and to face great barriers and higher costs in accessing healthcare [1]. Consideration therefore needs to be given as to how to include the large number of people with disabilities within ambitions to achieve UHC.

**Figure 1:**
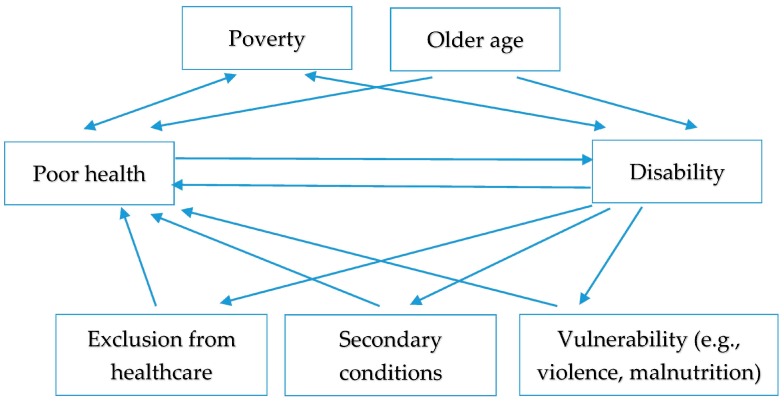
Complex relationship between disability and poor health.

Considering the relationship between health and disability is complex as these constructs are overlapping, inter-twined and reinforcing (Figure 1). Furthermore, people with disabilities are a highly diverse group, including people with a range of impairment types, age, gender and environments. The relationship between health and disability will therefore not be the same for all.

On the one side, poor health may lead to disability. People with disabilities, by definition, have an impairment as a result of an underlying health condition (Table 1) [1,14]. Disability is not the inevitable consequence of poor health or impairment but occurs in the context of unfavourable personal or environmental factors that hinder the person’s full and effective participation in society on an equal basis with others. A health condition or impairment is therefore a necessary condition for disability, but is not a sufficient cause on its own.

On the other side, disability may contribute to worsening health. The underlying health condition causing the disability may have other negative impacts, for instance diabetes may cause visual impairment but also kidney or nerve damage. The impairment may itself be associated with an increase in risk of poor health outcomes, for instance people with spinal cord injuries are at risk of developing pressure sores, or people with limited mobility are prone to osteoporosis. People with disabilities are also on average older [1] and therefore more likely to experience multiple health conditions at the same time. The unfavourable structural condition of people with disabilities, in terms of their poverty and exclusion, will also make them more vulnerable to ill health and injuries [15,16]. For instance, poverty is associated with malnutrition, inadequate access to public health services (e.g., immunisation), poor living conditions (e.g., lack of safe water) and environmental exposures (e.g., unsafe work environments) and so these issues will disproportionately affect people with disabilities [16]. People with disabilities may also experience barriers to accessing health care services, or inadequate quality of health care, which also potentially results in poorer health [1]. These links are not the same for all people with disabilities. For instance, people with intellectual impairments may face more exclusions from health promotion activities, while people with physical impairments may be particularly vulnerable to difficulties caused by physical accessibility.

A clear consequence of the vulnerability of people with disabilities to poor health is the need to ensure their good access to healthcare, and without their inclusion UHC is unlikely to be achieved. Access to health is also the fundamental right of people with disabilities, as set out in the UN Convention on the Rights of Persons with Disabilities [14]. Access should be to the full spectrum of services, addressing both general healthcare needs (e.g., sexual and reproductive health services) as well as impairment-focussed care (e.g., rehabilitation services). These services are needed to treat conditions as they arise, to prevent further morbidity and reduce mortality. Provision of curative and rehabilitation services alone is insufficient. Health promotion is also a priority to maintain health and avoid long-term health effects associated with disability. Health promotion should tackle both general and specialist health issues—acknowledging that people with disabilities require the same health promotion as people without disabilities (e.g., sexual health [17,18,19], oral health [20]) but may also require other specific messaging (e.g., prevention of secondary complications such as contractures) [3].

## 3. Health Promotion for People with Disabilities

Health promotion is defined by WHO as “*the process of enabling people to increase control over and to improve, their health*”. At the individual level, health promotion aims to promote health and healthy lifestyles through personal behaviour change. These interventions include promoting good nutrition, undertaking regular physical activity and engaging in vaccine and preventative health initiatives (e.g., vitamin A supplementation). There is strong evidence that people with disabilities may fall behind in terms of individual behaviours related to a healthy lifestyle, for instance, they are on average more likely to be physically inactive [6,10,21], smoke [6,10] and use illegal drugs [22,23]. Low engagement in preventative health behaviours is also of concern among people with disabilities [11,24,25,26]. As examples, people with disabilities are less likely to attend regular visits to the dentist [27] or take part in cancer screening [28,29], or be reached by messaging about HIV [30,31].

There is therefore an urgent need to focus on people with disabilities in health promotion activities, because they are at higher risk of poor health and are falling behind in individual healthy behaviours. This focus is currently not happening well for a variety of reasons. Information may not be transmitted in accessible formats, such as braille, sign-language or easy read. There may be misconceptions that people with disabilities do not need certain services, such as information about sexual health and they are therefore not targeted with these messages. People with disabilities may also be excluded from the health promotion campaigns for other reasons. For instance, children with disabilities, who are less likely to attend school, may not be reached with school-based health promotion activities. Pragmatic evidenced-based solutions are needed to overcome these barriers and better meet the needs of people with disabilities in health promotion [32,33].

A twin-track approach has been long recognised and advocated for within the disability community. This approach aims to ensure that people with disabilities are included in mainstream programming but also have specific targeted interventions to meet any additional needs (Examples given in Table 2). If we consider this approach with respect to health promotion for people with disabilities, it means first ensuring that they are included within mainstream health promotion activities (e.g., including examples of people with disabilities in health promotion material, providing material in braille, ensuring meetings are held at accessible locations) but also targeting people with disabilities with specific interventions around health promotion (e.g., providing people with mobility impairments information about how to prevent bed sores) [34,35,36,37,38,39]. People with disabilities have unique insights about their disability and situation, but are often excluded from the decision-making process about issues that directly affect their lives, ref. [40] and this must be addressed in planning health promotion activities, whether mainstream or targeted.

Health promotion also has a broader aim, however, beyond changing individual behaviours. This broader aim is to tackle the determinants of health, for instance through improving income, housing, food security, employment and quality working conditions. We have already described how people with disabilities are more likely to experience structural inequalities, such as exclusion from jobs and schooling and higher levels of poverty [1,2] and these factors will make them vulnerable to poor health. The broader ambition of health promotion is therefore to improve the inclusion and living conditions of people with disabilities in order to promote and preserve their health. This more holistic and far-reaching approach to health promotion for people with disabilities therefore has the same ambitions as the SDGs including: less poverty, better living standards and equitable inclusion in employment and education [3]. Again, a twin-track strategy can be used to address these inequalities, ensuring both that people with disabilities are included in mainstream programmes and that they are additionally targeted with specific interventions.

Targeting the drivers of poor health can be challenging, though ultimately is likely to have the biggest impact. As an example, people with disabilities often have difficulties in accessing Water, Sanitation and Hygiene (WASH) services [41], which will make them vulnerable to poor health. Providing accessible facilities alone will likely reap some benefits towards overcoming these barriers. However, making efforts to address stigma and discrimination and improving the policy framework around disability will likely improve not only access to WASH but will also have more wide-reaching impacts for people with disabilities, such as encouraging inclusion in school, jobs and society at large [41,42].

The focus of health promotion therefore needs to be expanded, if UHC is to be achieved. First, it needs to be inclusive of people with disabilities as they are a large and vulnerable group. Second, it needs to tackle the underlying drivers of poor health as well as immediate behaviours. Health promotion policies and activities should therefore address deeper causes of poor health, as well as health behaviours.

## 4. A Case Study on Health Promotion and Disability: Children with Congenital Zika Syndrome

We can make this discussion on health promotion and disability more concrete by considering the example of Congenital Zika Syndrome. Following the Zika Virus epidemic in South America in 2015, there was a massive increase in the number of babies born with microcephaly and other abnormalities, now collectively called “Congenital Zika Syndrome”. These children experience a range of health conditions, including severe developmental delay, intellectual and visual impairment and musculoskeletal abnormalities and epilepsy [43]. The affected children are also vulnerable to a range of secondary health conditions arising from the syndrome, such as respiratory illness, malnutrition and pressure sores. These health concerns will persist as the children transition into adulthood. Children with Congenital Zika Syndrome will therefore have a lifetime greater vulnerability to poor health and an increased need for a range of healthcare services as a result of their physical and intellectual impairments.

Promoting the health of these children is imperative. Health promotion messaging is mostly directed at parents, as they are usually the main carers for children with Congenital Zika Syndrome. Health promotion efforts focus on improving knowledge, skills and behaviours of parents so that they can (1) better address their child’s existing health issues (e.g., control of seizures) to prevent them causing further concerns; (2) avoid the occurrence of health problems in the future (e.g., malnutrition through better feeding, contractures through improved positioning) and (3) maximize the development of their child (e.g., through early stimulation). These efforts aim to preserve good health as far as possible, maximise development and quality of life and reduce the need of these children for healthcare services.

There is an important concern with this approach. Looking after a child with severe developmental disabilities and complex needs places an enormous emotional strain on families, with resultant high risks of paternal abandonment and maternal mental health concerns. Families of children with Congenital Zika Syndrome are disproportionately more likely to be poor and also often experience further financial strain through medical costs and lost income. Parents are therefore being expected to take on caring tasks for their child with complex needs, with little training or support, while they are also experiencing emotional distress, poverty and disadvantage. Developing interventions to improve the knowledge and skills of parents to change behaviours are unlikely to be effective and sustainable long-term without addressing these other difficulties that the parents may face. As a consequence, health promotion efforts need to go beyond improving the knowledge and behaviour of parents to also addressing other underlying determinants of poor health for these children: poverty, exclusion and carer distress.

One potentially effective strategy to achieve more holistic and sustainable health promotion is through caregiver support programmes [44]. We used this approach to develop a carer-support intervention targeting parents of children with Congenital Zika Syndrome in Brazil. This programme aims to support the families, both in terms of providing psychosocial support and in improving their skills, to be able to care for their child optimally and to connect to available services. The programme also focusses on the underlying drivers of poor health: poverty, stigma and exclusion. Ultimately, this programme aims to promote the health and functional status of the child and eventually the child’s (and the parents’) participation in society. Using the twin-track framework described above, this is an example of a targeted intervention and complements mainstream programmes that are inclusive of children with disabilities.

The health promotion intervention is offered through groups of parents and their children with Congenital Zika Syndrome. It consists of 10–11 sessions offered over a period of 3 months. The content of the programme includes information about essential care practices, such as feeding, positioning, communication, play and early stimulation, in order to promote health and maximise development. This focus takes learning from some of the health concerns that often affect children with cerebral palsy (a similar condition to Congenital Zika Syndrome) such as contractures and malnutrition and aims to preserve health and avoid the occurrence of these secondary health conditions. It also encourages effective health seeking behaviour and the sharing of experiences between parents as to how this can be achieved. For instance, in the food and nutrition module, there is a focus on what constitutes a balanced diet—important for all children. This module also covers positioning for feeding for children with disabilities and looking out for signs of aspiration—a potential risk for some children with developmental delays, which can lead to severe respiratory problems.

The programme goes beyond a focus on changing individual behaviours and promoting the child’s physical condition and health; It also addresses disability rights and how parents can advocate for their child’s inclusion in school and health care and receipt of disability benefits. As an example, navigating the health care system is an important concern raised by parents. A session in the programme helps parents to understand how they can get the most out of health visits, which may encourage them to attend appointments regularly and engage more effectively in their child’s care plan. The ambition is that the parent groups will become self-sustaining and a focus in the latter part of the programme is on how to run parent groups and engage effectively with the community to overcome stigma and discrimination and promote inclusion and acceptance. The emotional support activity, as part of every session, has helped to stimulate open and supportive discussion between parents about their successes and difficulties. This component fosters an atmosphere of empathy and solidarity, contributing towards mental health promotion for carers and their improved capacity to look after their child. The programme therefore also aims to empower parents so that they can address the drivers of poor health among their children with disabilities, which include stigma and discrimination, exclusion from health care services and poverty.

The parent groups are led by two facilitators—one therapist and one parent of child with Congenital Zika Syndrome (“expert mother”). The role of the expert mother is crucial in order to facilitate a participatory and egalitarian atmosphere and to encourage sharing of learning between parents. The facilitators attended a one-week training course before the start of the programme and are given on-going support through a set of materials, regular supervisory visits and access to mentors. Groups are held in the local community (e.g., local health centre or church) so that strong networks can be built between carers who live close to each other. The sessions are participatory and include activities, open discussions, explanations, demonstrations and light-hearted ice breakers (Table 3). This approach is informed by adult learning theory and aims to minimise issuing further instructions to over-loaded parents [45].

The intervention is currently undergoing pilot testing with 6 parent groups spread across Rio de Janeiro and Salvador to assess the feasibility and acceptability of the intervention. The next stage is to evaluate the effectiveness of the programme in improving the health (e.g., nutritional status, utilization of health care services) and functional status of children (e.g., measured using Bayley Scales of Infant Development) and the mental health (e.g., using PHQ-9) and quality of life of parents (e.g., using WHO Quality of Life scale), ideally through a randomized controlled trial.

## 5. Conclusions

The focus of health promotion needs to be expanded if UHC is to be achieved. First, health promotion activities must be inclusive of people with disabilities as they are a large group who are more vulnerable to poor health. In addition, health promotion must also address poverty and inequality as key drivers of poor health, since tackling individual behaviours alone is insufficient. Achieving inclusion of people with disabilities in health promotion will be helped if this is supported by appropriate policies and if evidence is generated as to how inclusion can be achieved. One potential approach is through parent support programmes which offer innovative and sustainable ways to improve the skills and knowledge of parents and thereby the health of their children with disabilities and the drivers of poor health (e.g., poverty, stigma) as part of a wider strategy. Including people with disabilities in health promotion will ultimately help to make sure that we Leave No-one Behind as we move towards Universal Health Coverage.

## Figures and Tables

**Table 1:** Definitions of Disability.

UN Convention on the Rights of Persons with Disabilities	Persons with disabilities include those who have long-term physical, mental, intellectual or sensory impairments which in interaction with various barriers may hinder their full and effective participation in society on an equal basis with others [14].
World Report on Disability	[Disability is] an umbrella term for impairments, activity limitations and participation restrictions, denoting the negative aspects of the interaction between an individual (with a health condition) and that individual’s contextual factors (environmental and personal factors) [1].

**Table 2:** Examples of the twin-track approach.

Issue	Mainstream Intervention	Targeted Intervention
Children with disabilities are excluded from school	Ensure that policies reinforce the rights of children with disabilities to education	Provide financial subsidies to children with disabilities to facilitate their school attendance
People with disabilities are more likely to be poor	Ensure that people with disabilities are eligible for social protection programmes	Offer vocational training for people with disabilities
Women with disabilities receive inadequate sexual health services	Train doctors on the needs of women with disabilities for these services	Provide information to women with disabilities about their right to healthcare and how they can realise these rights

**Table 3:** Example of part of a facilitated group session on eating and drinking.

Example	Discussion	Aim
**Ice-breaker** In pairs: One person tries to give the other a drink of water in different positions (e.g., head leaning back, turned to one side, or flopping forwards).	*How easy or difficult is it to swallow in each position? How does it feel to be fed?*	To understand a range of issues that your child may experience with eating and drinking.
**Discussion** As a large group to share experiences	*What is a nutritious or “balanced” diet?*	To know what a balanced diet is and how to maximise your child’s nutritional intake and prevent malnutrition.
**Activity** Show a banana and a biscuit and other common foods.	*Discuss—Are the items hard or soft? Can they be made into a smooth puree? How?*	To learn ways to feed your child safely

## References

[1] World Health Organisation (2011). World Report on Disability.

[2] Banks L.M., Polack S. (2014). The Economic Costs of Exclusion and Gains of Inclusion of People with Disabilities.

[3] United Nations Sustainable Development Goals. http://www.un.org/sustainabledevelopment/sustainable-development-goals/.

[4] Mactaggart I., Kuper H., Murthy G.V., Sagar J., Oye J., Polack S. (2016). Assessing health and rehabilitation needs of people with disabilities in Cameroon and India. Disabil. Rehabil..

[5] Kuper H., Monteath-van Dok A., Wing K., Danquah L., Evans J., Zuurmond M., Gallinetti J. (2014). The impact of disability on the lives of children; cross-sectional data including 8900 children with disabilities and 898,834 children without disabilities across 30 countries. PLoS ONE.

[6] Froehlich-Grobe K., Jones D., Businelle M.S., Kendzor D.E., Balasubramanian B.A. (2016). Impact of disability and chronic conditions on health. Disabil. Health J..

[7] Stevens A., Courtney-Long E., Gillespie C., Armour B.S. (2014). Hypertension among US adults by disability status and type, National Health and Nutrition Examination Survey, 2001–2010. Prev. Chronic Dis..

[8] Kinne S., Patrick D.L., Doyle D.L. (2004). Prevalence of secondary conditions among people with disabilities. Am. J. Public Health.

[9] Wilber N., Mitra M., Walker D.K., Allen D., Meyers A.R., Tupper P. (2002). Disability as a public health issue: Findings and reflections from the Massachusetts survey of secondary conditions. Milbank Q..

[10] AIHW Health of Australians with Disability: Health Status and Risk Factors. http://www.aihw.gov.au/publication-detail/?id=6442472401.

[11] Reichard A., Stolzle H., Fox M.H. (2011). Health disparities among adults with physical disabilities or cognitive limitations compared to individuals with no disabilities in the United States. Disabil. Health J..

[12] Altman B.M., Bernstein A. (2008). Disability and Health in the United States, 2001–2005.

[13] Rotarou E.S., Sakellariou D. (2017). Neoliberal reforms in health systems and the construction of long-lasting inequalities in health care: A case study from Chile. Health Policy.

[14] United Nations (2006). Convention on the Rights of Persons with Disabilities.

[15] Groce N., Kett M., Lang R., Trani J.-F. (2011). Disability and Poverty: The need for a more nuanced understanding of implications for development policy and practice. Third World Q..

[16] Mitra S., Posarac A., Vick B. (2013). Disability and Poverty in Developing Countries: A Multidimensional Study. World Dev..

[17] Kassa T.A., Luck T., Bekele A., Riedel-Heller S.G. (2016). Sexual and reproductive health of young people with disability in Ethiopia: A study on knowledge, attitude and practice: A cross-sectional study. Glob. Health.

[18] Best K. (1999). Disabled have many needs for contraception. Netw. Res..

[19] Trani J.-F., Browne J., Kett M., Bah O., Morlai T., Bailey N., Groce N. (2011). Access to health care, reproductive health and disability: A large scale survey in Sierra Leone. Soc. Sci. Med..

[20] Arunakul M., Kuphasuk Y., Boonyathanasit R. (2012). Effectiveness of oral hygiene instruction media on periodontal health among hearing impaired children. Southeast Asian J. Trop. Med. Public Health.

[21] Ko K.D., Lee K.Y., Cho B., Park M.S., Son K.Y., Ha J.H., Park S.M. (2011). Disparities in health-risk behaviors, preventive health care utilizations and chronic health conditions for people with disabilities: The Korean National Health and Nutrition Examination Survey. Arch. Phys. Med. Rehabil..

[22] Gilson S.F., Chilcoat H.D., Stapleton J.M. (1996). Illicit drug use by persons with disabilities: Insights from the National Household Survey on Drug Abuse. Am. J. Public Health.

[23] Glazier R.E., Kling R.N. (2013). Recent trends in substance abuse among persons with disabilities compared to that of persons without disabilities. Disabil. Health J..

[24] Chun S.M., Hwang B., Park J.H., Shin H.I. (2012). Implications of sociodemographic factors and health examination rate for people with disabilities. Arch. Phys. Med. Rehabil..

[25] Diab M.E., Johnston M.V. (2004). Relationships between level of disability and receipt of preventive health services. Arch. Phys. Med. Rehabil..

[26] Iezzoni L.I., McCarthy E.P., Davis R.B., Siebens H. (2000). Mobility impairments and use of screening and preventive services. Am. J. Public Health.

[27] Oredugba F.A., Perlman S.P. (2010). Oral health condition and treatment needs of Special Olympics athletes in Nigeria. Spec. Care Dentist..

[28] Horner-Johnson W., Dobbertin K., Lee J.C., Andresen E.M. (2013). Disparities in chronic conditions and health status by type of disability. Disabil. Health J..

[29] Floud S., Barnes I., Verfurden M., Kuper H., Gathani T., Blanks R.G., Alison R., Patnick J., Beral V., Green J. (2017). Disability and participation in breast and bowel cancer screening in England: A large prospective study. Br. J. Cancer.

[30] Groce N.E., Yousafzai A.K., van der Maas F. (2007). HIV/AIDS and disability: Differences in HIV/AIDS knowledge between deaf and hearing people in Nigeria. Disabil. Rehabil..

[31] UNAIDS (2014). The Gap Report.

[32] Reich M.R., Harris J., Ikegami N., Maeda A., Cashin C., Araujo E.C., Takemi K., Evans T.G. (2016). Moving towards universal health coverage: Lessons from 11 country studies. Lancet.

[33] Coe G., de Beyer J. (2014). The imperative for health promotion in universal health coverage. Glob. Health Sci. Pract..

[34] Otte W.M., van der Maas F., de Boer A. (2008). Comparison of knowledge and accessibility to information sources of HIV/AIDS between blind and sighted populations in Nigeria. AIDS Care.

[35] Bisol C.A., Sperb T.M., Brewer T.H., Kato S.K., Shor-Posner G. (2008). HIV/AIDS knowledge and health-related attitudes and behaviors among deaf and hearing adolescents in southern Brazil. Am. Ann. Deaf..

[36] Eide A.H., Schur C., Ranchod C., Rohleder P., Swartz L., Schneider M. (2011). Disabled persons’ knowledge of HIV prevention and access to health care prevention services in South Africa. AIDS Care.

[37] Smeltzer S.C., Zimmerman V., Frain M., DeSilets L., Duffin J. (2004). Accessible online health promotion information for persons with disabilities. Online J. Issues Nurs..

[38] Morrison J., Colbourn T., Budhathoki B., Sen A., Adhikari D., Bamjan J., Pathak S., Basnet A., Trani J.F., Costello A. (2017). Disabled women’s attendance at community women’s groups in rural Nepal. Health Promot. Int..

[39] Ravesloot C., Ruggiero C., Ipsen C., Traci M., Seekins T., Boehm T., Ware-Backs D., Rigles B. (2011). Disability and health behavior change. Disabil. Health J..

[40] Khan F., Owolabi M.O., Amatya B., Hamzat T.K., Ogunniyi A., Oshinowo H., Elmalik A., Galea M.P. (2017). Challenges and barriers for implementation of the World Health Organization Global Disability Action Plan in low- and middle- income countries. J. Rehabil Med..

[41] Groce N., Bailey N., Lang R., Trani J.F., Kett M. (2011). Water and sanitation issues for persons with disabilities in low- and middle-income countries: A literature review and discussion of implications for global health and international development. J. Water Health.

[42] White S., Kuper H., Itimu-Phiri A., Holm R., Biran A. (2016). A Qualitative Study of Barriers to Accessing Water, Sanitation and Hygiene for Disabled People in Malawi. PLoS ONE.

[43] Moore C.A., Staples J.E., Dobyns W.B., Pessoa A., Ventura C.V., Fonseca E.B., Ribeiro E.M., Ventura L.O., Neto N.N., Arena J.F. (2017). Characterizing the Pattern of Anomalies in Congenital Zika Syndrome for Pediatric Clinicians. JAMA Pediatr..

[44] Collins P.Y., Pringle B., Alexander C., Darmstadt G.L., Heymann J., Huebner G., Kutlesic V., Polk C., Sherr L., Shih A. (2017). Global services and support for children with developmental delays and disabilities: Bridging research and policy gaps. PLoS Med..

[45] McConachie H., Huq S., Munir S., Kamrunnahar, Akhter N., Ferdous S., Khan N.Z. (2001). Difficulties for mothers in using an early intervention service for children with cerebral palsy in Bangladesh. Child Care Health Dev..

